# Assessment of Childhood Immunisation Services in Kano and Lagos States, Nigeria: A Mixed-methods Study

**DOI:** 10.1177/11786329261477836

**Published:** 2026-09-17

**Authors:** Adekunle Ganiyu Salaudeen, Oladimeji Akeem Bolarinwa, Catherine R. McGowan, Drichi Daniel, Mervat Alhaffar, Kaja Abbas, Olatunde Adesoro, Tahlil Ahmed, Andrew Clarke, Sostine Makunja, Nada Abdelmagid, Tanimola Makanjuola Akande

**Affiliations:** 1 Department of Epidemiology and Community Health, 108285 University of Ilorin, Ilorin, Nigeria; 2 Department of Infectious Disease Epidemiology and International Health, 170945 London School of Hygiene and Tropical Medicine, London, UK; 3 Department of Infectious Disease Epidemiology and Dynamics, 4906 London School of Hygiene and Tropical Medicine, London, UK; 4 School of Tropical Medicine and Global Health, Nagasaki University, Nagasaki, Japan; 5 663101 BOOST Project Team, Save the Children International, Abuja, Nigeria; 6 Global Health Team, Save the Children UK, London, UK

**Keywords:** immunisation, zero-dose, under-immunisation, Nigeria, children, equity

## Abstract

**Background:**

Recent evidence suggests that just over a third of children aged 12–23 months in Nigeria have received *basic* antigens as recommended by the expanded programme on immunisation schedule; just over a quarter have received *all* first year antigens. Approximately 18% of children in Nigeria are classified as ‘zero-dose’. We assessed the status of immunisation services in Kano and Lagos States of Nigeria to inform the design of interventions to address high numbers of zero-dose and under-immunised children in these states.

**Methods:**

We used a mixed-methods study design. We collected information from caregivers (including female and male caregivers; and caregivers of zero-dose, under-immunised, and immunised children), and healthcare workers. We conducted focus groups and key informant interviews amongst caregivers of zero-dose and non-zero-dose children. We interviewed caregivers and observed vaccination sessions. We carried out facility observation of vaccination sessions. Data collection tools were adapted from the WHO expanded programme on immunisation review guidelines.

**Results:**

Whilst we identified many positive practices - and exit interviews suggested high satisfaction with vaccination services - we identified important areas for improving services. Specific challenges related to the: 1) staff (including insufficient staff to operate vaccination activities, poor information exchange with caregivers, low planning capacity, sub-optimal health and safety practices, and abrasive healthcare worker attitudes); 2) facility (including unwelcoming and uncomfortable facility environments and occasional vaccine shortages), and 3) programme design/delivery (including poor planning/service integration, and insufficient resources for transportation/outreach services).

**Conclusion:**

We suspect that many of the identified challenges are interrelated (e.g. insufficient staff numbers, and an uncomfortable facility environment, may contribute to poor interaction between healthcare workers and caregivers) and that addressing some of these challenges may have cascading effect on vaccination coverage. A better understanding of improving service design and delivery to incentivise visits to vaccination services is needed.

## Introduction

Despite a significant reduction in child mortality between 1990 and 2015, sub-Saharan Africa is still characterised by high mortality amongst children under-five years of age. As of 2022, the under-five mortality rate (U5MR) in the region was 71 deaths per 1000 live births.^1^ Approximately one in five children in the region do not receive basic vaccines and over half a million children under-five die from vaccine preventable diseases (VPDs) annually, equating to 58% of global VPD-related deaths.^2^ Vaccination is recognised as a cost-effective public health measure for decreasing childhood mortality and morbidity.^2^ Routine immunisation offers one of the highest returns on investments in health, saving up to $44 USD in additional benefits for every $1 USD spent.^2^

Globally, more than 50% of zero-dose (ZD) children – i.e. those children not receiving their first dose of diphtheria, pertussis, and tetanus vaccine (DTP1) – are found in seven countries: Nigeria, India, Ethiopia, Democratic Republic of the Congo (DRC), Sudan, Indonesia, and Yemen.^2^ Eighteen percent of children in Nigeria are classified as ZD.^3^ A major survey finding from the 2021 Multiple Indicators Cluster Survey/National Immunization Coverage Survey Report for Nigeria reveals that amongst children aged 12-23 months, just over a third (36%) had received all basic antigens (i.e. BCG, OPV 1-3, DTP 1-3 and Measles 1) by 12 months, as recommended by Nigeria’s routine immunisation schedule.^3^

Both ZD and under-immunisation (UI) - typically defined as those children not receiving their third dose of DTP (i.e. DTP 3) -, are thought to share some key risk factors including: maternal factors, access to ante-/perinatal care, location, religious background, wealth, religion, population composition, proximity of facilities offering vaccination, and family awareness about immunisation (including safety concerns).^4,5^ The COVID-19 pandemic has also had a considerable impact on childhood vaccination in Nigeria; an estimated 6.2 million children did not receive a single vaccine dose between 2019 and 2022 due to the pandemic.^6^ To reach these vulnerable children with life-saving vaccines, the government of Nigeria rolled out the ‘Big Catch-up Campaign’ which is aimed at reaching those children who missed-out on vaccination due to service disruptions.^6^

A 2022 review identified many risk factors for low childhood vaccination coverage in Nigeria, including many health system factors relating to service quality.^4^ Health system factors - including ineffective management, limited funding, negative attitudes of health facility staff, overcrowded facilities, low numbers of healthcare workers (HCW), and poor-quality services – often disincentivise vaccination uptake.^4,7^

Regular assessment of immunisation services is important for providing constant feedback to service providers and managers about gaps, barriers, and opportunities. Addressing gaps and barriers, and taking advantage of opportunities have the potential to strengthen immunisation service quality, which is key to increasing vaccination coverage and reducing drop-out.^8^ Therefore, to inform the design of a package of interventions to address low vaccination uptake in regions with high numbers of ZD and UI children, we carried out an immunisation assessment to describe the quality of vaccination services (both from the perspective of service providers, and service users) in Kano and Lagos States, Nigeria.

## Methods

### Aim and Objectives

The study aimed to describe the quality of current immunisation services and inform programme intervention design in selected sites in Kano and Lagos States. The objectives were to: 1) understand the existing service provision, 2) identify best practices and challenges driving ZD and UI, and 3) develop recommendations for improving vaccination coverage in Nigeria.

### Study Setting

This study was carried out in Kano and Lagos States of Nigeria. Kano and Lagos States are both urban states with high volume commercial activities, though both states have rural components. The study sites were purposively selected due to the large number of resident ZD and UI children. Kano is predominantly occupied by people of Islamic faith, whilst Lagos is a mixture of Muslims and Christians as well as some traditional religion practitioners. Kano State is the largest state by population in Nigeria with an estimated population of 13 million in 2016.^9^ It is estimated that despite recent improvements, 23% of children in Kano State were ZD in 2022; drop-out was estimated to be 5% in 2023.^10^ Lagos State is the second most populous state with an estimated population of 12.5 million.^9^ Alimosho local government area (LGA) in Lagos State has the highest absolute number of ZD children in Nigeria with more than 35, 000 ZD children.^11^ We focussed on two LGAs in each state: Ungogo and Gezawa in Kano State, and Alimosho and Ikorodu in Lagos State. The LGAs were chosen because of their high burden of ZD children. Six health facilities in each LGA were selected for the study for a total of 24 facilities across the two states.

### Study Design

We used a mixed-methods approach to collect data from caregivers and HCWs. We developed qualitative and quantitative tools based on the WHO guidance on conducting EPI reviews and the behavioural and social drivers (BeSD) framework for vaccination.^12,13^ The data collection method, study tools, and study populations are included in Table 1.

**Table 1.** Data Collection Method, Data Collection Tools, and Study Population

​	Healthcare workers (HCW)		Caregivers			
Data collection method	Facility observation	Key informant interviews (KII)	Focus group discussion (FGD)		Exit interviews	
Tool	Vaccination session observation form^12^	Health facility level questionnaire^12^	Focus group discussion guide (informed by^13^)		Caregiver Exit Interviews Form^12^	
Study population	Administered in the health facility during vaccination sessions	HCWs involved in immunisation services (with >3 month’s experience in delivering immunisation services)	Caregivers with ZD children < 2 years of age	Caregivers with no ZD children < 2 years of age	Caregivers aged between 15-17	Caregivers aged 18+

### Sampling and Recruitment

#### Healthcare Workers

Healthcare worker study participants were selected purposively (based on their role in immunisation activities) to participate in key informant interviews (KIIs). The health facilities holding ongoing immunisation sessions were selected for facility observation.

### Caregivers

Eligible caregivers attending immunisation sessions in each of the included health facilities were invited to participate in an exit interview. For the focus group discussions (FGDs) caregivers were divided into two groups: those with zero-dose children, and those with non-zero-dose children (both based where possible, on vaccination card information). We selected FGD participants with the aim of ensuring representation; as such, we included young mothers (<18 years of age), and both male and female caregivers. Each FGD had between 8 and 12 participants, depending on the location.

### Data Collection and Analysis

Facility observation and exit interview data were collected via ODK (ODK: v 1.3.2 [client], v 1.3.3 [server] in March/April 2024. San Diego, CA: Get ODK Inc); descriptive statistics were generated using STATA (StataCorp. 2023. Stata Statistical Software: Release 18. College Station, TX: StataCorp LLC). All open-ended questions from the interviews were transcribed verbatim. Qualitative data obtained from the FGDs and KIIs were audio recorded; notes were taken during the interviews. The audio-recorded data were transcribed and translated and were de-identified using unique identification numbers. We de-identified participants at the point of transcription. The transcripts were coded using a thematic approach (see Table 2). Themes were based on the topics, and sub-topics of the WHO EPI review guidelines.^12^ NVivo software (QSR International Pty Ltd.) version 12 Plus, 2018 was used to organise the data, which were summarised into overarching themes using a reflexive thematic framework approach. The phases of this approach are described below. Phase 1 included data management for which there were three steps; familiarisation, generating initial codes, and indexing. Phase 2 involved defining themes. We sorted the codes into themes and organised them into broader themes. We then linked the themes to the main research aim.

**Table 2.** Coding Topics and Sub-topics Included in the WHO Guide for Conducting an Expanded Programme on Immunization (EPI) Review

Topic	SUB-TOPICS
1. Programme management and financing	A. Policy and guidance B. Governance and accountability C. Planning and procurement D. Partner coordination E. Budgeting and financing
2. Human resources management	A. HR planning B. Capacity-building C. Supervision and performance monitoring
3. Vaccine supply, quality, and logistics	A. Cold chain B. Supply management C. Transport D. Waste management
4. Service delivery	A. HR and strategies B. Session quality C. Integration
5. Immunisation coverage and adverse event following immunisation (AEFI) monitoring	A. HR and systems B. Recording and reporting C. Data quality D. Coverage monitoring and use E. AEFI monitoring
6. Disease surveillance	A. HR and systems B. Detection and response C. Performance
7. Demand generation	A. Demand B. Advocacy and communication C. Community engagement

We held KIIs with 55 HCWs in the two states (28 in Kano, and 27 in Lagos). Interview participants included: senior facility staff and immunisation-related staff (including vaccinators, cold-chain technicians, volunteers, and others involved in outreach services). We carried out 18 FGDs (12 in Kano, and six in Lagos) in the health facility catchment areas. The FGDs included caregivers with ZD, UI, and fully vaccinated children. We selected participants to ensure diversity; including young mothers (<18 years of age), and both male and female caregivers.

Exit interviews were conducted with 611 caregivers (302 in Kano, and 309 in Lagos). The facility observation checklist was completed for 32 facilities: 12 in Kano, and 20 in Lagos. The number of participants recruited for each data collection activity are presented in Table 3.

**Table 3.** Number of Facility Observations, Exit Interview Participants, Focus Groups, and Key Informant Interviews

​	Quantitative data		Qualitative data	
Local government areas (State)	Facility observation (# of facilities)	Exit interviews (# of caregivers)	Focus group discussions (FGD)	Key informant interviews (KII)
Gezawa (Kano)	7	155	12	28
Ungogo (Kano)	5	147		
Alimosho (Lagos)	14	156	6	27
Ikorodu (Lagos)	6	153		
Total	32	611	18	55

### Ethics Approval

Ethics approval was obtained from the National Health Research Ethics Committee of Nigeria (NHREC/01/01/2007-04/12/2023) and the University of Ilorin (UERC/ASN/2024/2673). Similarly, ethics approval was obtained in Kano State (SHREC/2023/4555), whilst social approval was secured in Lagos State (LSMH/6211/vol1/80). Ethics approval was also obtained from the London School of Hygiene and Tropical Medicine’s Research Ethics Committee (REC# 29802). Written informed consent was obtained from KIIs, FGDs, and exit interview participants. We conducted this study in accordance with the Helsinki Declaration of 1975, as revised in 2024.

## Results

### Basic Immunisation Topics

We report our findings below against the basic immunisation topics in the WHO guide for conducting an EPI review.^12^ All quantitative findings, disaggregated by LGA, are presented in **Additional file 1** (facility observation), and **Additional file 2** (exit interviews). Suggestions from facility observations and caregivers for addressing service design and delivery challenges are included in **Additional file 3**. All additional files are included in the Supplemental Material.

#### Programme Management and Financing

The KIIs revealed that some facilities in Kano State have operational guidelines (e.g. immunisation schedules and policies, facility-based micro-plans for routine immunisation, maps of catchment areas). However, KIIs suggest that HCWs lack sufficient tools to estimate the population of <1 year old children(e.g. to inform service planning, supply forecasting), and to prepare budgets. Furthermore, many HCWs indicated that their facilities do not receive financial support from the government for routine immunisation (RI) services (non-governmental organisations [NGOs] provide some limited funds for EPI programs) or for specific immunisation activities.

*“…in the past, it was given, we used to get 4, 000 naira, but they have stopped giving it out now. [The money] was for outreach service in the rural areas. We don’t have any budget for immunisation activities in this facility; we don’t have any money at the facility level for routine immunisation activities”.*

*(KII, HCW, Kano)*

#### Human Resources Management

During facility observations between one and five HCWs were typically present during vaccination sessions in both states. In Kano two (17%) of the 12 facilities were observed to have inadequate staffing numbers (this is defined, as per the operational definition used in Nigeria, as fewer than two health care workers trained to administer injectable vaccines present at a vaccination session involving 20 or more clients) for immunisation sessions. A more severe shortage of HCWs was observed during immunisation sessions in Lagos State where 12 (60%) of the 20 health facilities visited did not have enough staff present. The KIIs corroborated these observations revealing that there were between two and three HCWs (including recorders and vaccinators) providing immunisation services on any given day. Exit interviews suggested that staffing shortages were infrequent; however, caregivers may not be aware of staffing requirements for immunisation activities. However, despite staff shortages, most KII participants reported remaining dedicated to their duties, regardless of the circumstances.

*“In the immunisation unit, we are three. One person serves as the recorder, and two persons vaccinate children. The recorder is responsible for filling out all the data tools. We need additional manpower in the unit because the workload is too much; for example, the recorder fills out the vaccination card in the different data tools like the Voluntary Community Mobilizer (VCM). It would be good if there were two or three staff members doing that. And for us, the vaccinators, we are only two, if one of us did not come to work for any reason it is only one person that is left to do the work”.*

*(KII, HCW, Kano [Gezawa])*

To cushion the effect of understaffing some facilities employed the services of volunteer staff, such as community health extension workers (CHEWs), community health officers, and students undergoing practical training at the facility. It was also sometimes necessary to request support from HCWs working at nearby facilities. The number of volunteers (who assist HCWs) was higher in Kano State than Lagos State. Some participants in Kano and Lagos believed that three RI staff members would be sufficient to manage the facility’s immunisation services, given their dedication to their roles.

*“We are the only three workers working here. So as soon as the job allocation is done, we all work hand in hand to get the work done. The three workers here are the OIC [Officer-in-charge], the nursing officer, and the CHEW”.*

*(KII, HCW [nursing officer], Lagos [Alimosho])*

*“We are three in number; one of us is charged with the responsibility of filling out the registers and the data tool, and the remaining two people are vaccinating the children. I would say that they are enough”.*

*(KII, HCW [ward focal person], Kano [Gezawa])*

Finally, the KIIs revealed that in-service trainings are inadequate in areas such as planning, data management, and surveillance. During supportive supervision visits, HCWs reported receiving adequate on-the-job training and supervision. Ultimately, a significant number of health facilities face considerable staff shortages, and sometimes irregular payment of HCW salaries was reported to be demotivating.

#### Vaccine Supply, Quality and Logistics

Facility observations revealed that all health facilities in Lagos State stored vaccines at an appropriate temperature, with a single exception (in Gezewa, Kano State). Safety boxes were available in the majority of health facilities in both states. Syringe disposal (using the safety box) after vaccination was better (i.e. syringes were more often disposed of in a safety box after each vaccination) in Kano State (100%) than Lagos State (94%). All health facilities were observed to have safe sharp disposal equipment. In Kano State, 92% of health facilities reported appropriate waste disposal and management compared with 89% in Lagos State (waste management was not limited to syringe disposal but included all medical waste associated with the provision of vaccines). The KIIs suggest that HCWs receive technical support from trained cold chain officers.

Additionally, HCWs indicated that their facilities nearly always had sufficient vaccines to carry out RI. In the rare event of a stock-out HCWs noted that it was possible to obtain vaccines from another facility nearby.

*“I can’t even remember the last time we had a stock out of vaccines. But rarely, we may have one dose that is not available. When a dose is not available, we do go to a neighbouring health facility to collect a dose, like you just saw someone come on a bike to collect a dose here a while ago”.*

*(KII, HCW, Kano [Gezawa])*

*“The government is really trying from the aspect of immunisation - from the local government level to the health facility level - by making sure that all the vaccines and antigens are available in our health facility at all times, there was no time we had a shortage of vaccines, especially this year when we have all the antigens and we have a very good turnout of mothers for this immunisation. It is very important”.*

*(KII, HCW [OIC], Lagos [Ikorodu])*

A small number of HCWs reported inadequate transport mechanisms for vaccines to reach the service delivery point, and some HCWs reported using their personal funds for vaccine transportation.

*“…sometimes they will call us from [the vaccine delivery point] to go to collect. We may be very busy at the facilities so we have to write notes and send a bike person to go and collect for us. We just write the number of vaccines we require and give it to a bike man, to take it to where they are doing the distribution”.*

*(KII, HCW, Kano [Gezawa])*

#### Service Delivery

Facility observations revealed that all health facilities, in both states, administered vaccines appropriately, through the correct routes, and mixed or constituted them with the correct vaccine diluent. The staff in Kano State were perceived to be more organised and polite (92%), compared to those in Lagos State (89%). Similarly, staff in Kano also provided complete information about: 1) the vaccines that were administered to the children (100% in Kano, compared to 78% in Lagos), 2) the next vaccine to be received (100% in Kano, compared to 72% in Lagos), and 3) potential side effects (83% in Kano, compared to 67% in Lagos).

Nearly all caregivers knew the next vaccination appointment date in both states (98% in Kano and 96% in Lagos). Caregivers in both states indicated that the staff skills were optimal. Safe injection practices amongst staff were low in Kano compared to Lagos with 25% of staff observed touching or recapping syringes after use in Kano, compared to only 6% in Lagos. Both caregivers (during FGDs) and HCWs (during KIIs) reported that HCWs provide sufficient information about AEFIs and about follow-up appointments.

During the FGDs, male caregivers were asked for their views about immunisation; many of them agreed that vaccines are safe and effective. Some raised the issue of side effects, but felt that this was an acceptable trade-off as children were better off after vaccination. These views were expressed by male caregivers who supported vaccination and allowed their children to be vaccinated.

*“The vaccines are very safe to be taken by our children. Because we have brought our children and we have seen the good effect of the vaccines in comparison with other children that did not take the vaccines”.*

*(FGD, Fathers, Kano [Gezawa])*

*“It is safe. It is just that it may be hurtful and makes some children have their legs swollen. But as soon as it heals, then that is okay”.*

*(FGD, Fathers, Lagos [Ikorodu])*

In addition, many HCWs indicated that there is a clear immunisation strategy for reaching remote locations through outreach sessions; “*…any settlement that is beyond three kilometres from the facility, we choose it for outreach session because they are too far from the facility. Some people may not have money to travel to the facility*”. (KII, HCW, Kano).

There was evidence of limited integration of immunisation services with other essential health services. In Lagos many caregivers (86%) reported receiving growth monitoring in addition to vaccination services, but little else. In Kano nearly all respondents received only vaccination services despite evidence from KII suggesting that there are other services available on days when immunisation services are offered.

*“We do ante-natal care (ANC) services on the day we do routine immunisation. When they come for ANC, they see that children are brought for immunisation, so, during health talk, we do tell them about the benefits [of immunisation]. They see example with women they know who have come to get their babies immunised so that they too should do the same”.*

*(KII, HCW [OIC], Kano [Gezawa])*

The current service delivery system includes the tracking of RI defaulters. The current process involves taking photos of caregivers and their children during the first RI visit. If caregivers default, HCWs use the photographs to trace defaulters with support from community leaders. Additionally, HCWs typically request the phone numbers of caregivers, allowing them to send reminders, or to follow up with defaulters via text messages or phone calls. In Gezawa, Kano State HCWs noted that VCMs and traditional birth attendants (TBAs) are also involved in tracking defaulters.

*“In the past, we usually checked our register to see who missed their appointment, but with the coming of the new incentives, tracking defaulters has become so much easier for us because they take pictures of every mother and her child after vaccination. So once their system detects a defaulter, it brings out the mothers’ and the child’s picture together with their address. What we do is just go to that community and show the picture to the village head, and instantly he will direct us to her house. Then we explain to her that her child has missed a dose and we are here to vaccinate him/her”.*

*(KII, HCW, Kano [Gezawa])*

In Lagos State defaulter tracking is typically carried out through outreach sessions.

*“For tracking of defaulters, we do that through community outreaches. There is a session done in our facility every Wednesday as community outreach. So, we go into the community, especially in remote areas that we know might make it difficult for the people there to access the facility. We go to immunise the children in the comfort of their abode, and this is done weekly […]. We go from one community this week to another community the next week. There is a four-week interval before we visit a community again for immunisation”.*

*(KII, HCW [OIC], Lagos [Alimosho])*

Cancellations of immunisation sessions are a significant barrier to vaccination. For example, a HCW from Gezawa (Kano State) noted that the health facility had cancelled immunisation outreach sessions. Cancellations were attributed to a lack of funds for transportation. Outreach sessions were also cancelled in Ikorodu (Lagos State) due to a lack of funding and insufficient numbers of community mobilisers.

Caregiver exit interviews in Kano State also revealed that health facilities were sometimes not clean, waiting rooms were excessively hot, and that shaded waiting areas are not available. Caregivers also noted that there was an insufficient number of chairs at facilities, and no weighing scale.

#### Immunisation Coverage and AEFI Monitoring

All children who were vaccinated during facility observations had vaccination cards. Nearly all vaccination cards (>90%) were up to date for children in both states. All data tools (vaccine registers, vaccination cards, and tally sheets) were available in all facilities. The KIIs indicated that a significant number of HCWs had received training on the use of vaccination data tools. A focal person employed by the LGA supports data management and validates data collection monthly. Additionally, the government updated the data collection tools to incorporate newly introduced vaccines. Finally, health facility staff report their EPI data to the LGA and receive timely feedback.

Regardless, many HCWs do not use the data they collect to plan vaccination sessions. Moreover, some health workers reported that data entry is cumbersome and time-consuming.

*“We have so many registers for those immunisation service, and that is how we always strain ourselves to fill all of them. As I told you we have only one recorder and the number of data tools is many. If a child is brought for the first time, if it is not revisited, we enter the details in a card, we register him and vaccinate him, we can take like 15 minutes on each child”.*

*(KII, HCW, Kano [Gezawa])*

The fear of side effects after vaccination prevented some male caregivers from agreeing to vaccinate their children.

*“Usually, the pain that makes the child cry, as well he has a swollen leg for one to two weeks without having any relief. A child will cry for some time; that’s what prevents us from coming back to access the remaining vaccines”.*

*(FGD, Fathers, Lagos [Alimosho])*

*“I will use myself as an example, after immunising the baby, the baby was restless and couldn’t sleep properly for two days. Then I instructed my wife that she should never take my children for immunisation again”.*

*(FGD, Fathers, Kano [Gezawa])*

#### Disease Surveillance

A significant number of HCWs do not know the case definitions of acute flaccid paralysis (AFP), measles, and AEFI. Additionally, there is a lack of understanding of outbreak responses for notifiable diseases. Local government areas also delay providing feedback on vaccine-preventable disease surveillance results. Some health facilities lack AEFI kits, and most staff members lack the knowledge to carry out AEFI surveillance. For instance, when asked to describe the appropriate course of action in the event of an AEFI, HCWs were unable to provide a sufficiently accurate response.

#### Demand Generation

Exit interviews with caregivers revealed that a higher percentage of caregivers in Kano State (11%) had previously refused vaccinations, compared to 6% in Lagos State. More caregivers in Lagos State (15%) expressed concerns about vaccine safety, compared to caregivers in Kano State (9%). More than a third of caregivers (39%) in Kano State had difficulty accessing healthcare facilities, compared with just below a quarter (23%) in Lagos State. Almost all the caregivers in Kano State were satisfied with vaccination (99%) and all indicated that if they had another baby they would wish to have him/her vaccinated (100%). Similarly, 93% of caregivers were satisfied with vaccination and 99% indicated that if they had another baby they would wish to have him/her vaccinated in Lagos State. In this study, we observed that there were no gender preferences in vaccination; parents appear to make the decision to vaccinate their child independent of their child's gender.

*“I don't accept vaccination whether on male or female child* ***”.***

***(*** *FGD, Father, Kano [Gezawa])*

In Kano State 12% of caregivers had been turned away from vaccination in the past, whilst 10% had been turned away in Lagos State. The most common reason for turning away children from vaccination across both states was a shortage of vaccines. In Gezewa (Kano State) caregivers had also been turned away because the child was unwell and thus not deemed a candidate for vaccination, as well as due to staff shortages. In both LGAs in Kano State, children had been refused vaccination as the child was not the correct age.

## Discussion

Our research findings suggest that service quality was generally good; however, key areas of concern are discussed below.

### Factors Relating to Healthcare Workers

#### Poor Communication about AEFIs

Poor communication around AEFI is likely a consequence of the generally poor understanding of AEFI amongst HCWs. Our finding of poor HCW knowledge of AEFI was similarly observed in a recent study in Northwest Nigeria which concluded that the knowledge level around AEFI amongst HCWs in Kebbi State was, “varied and suboptimal”^14(p. 1)^. Despite gaps in AEFI understanding and communication, all HCWs in Kano State and in Alimosho (Lagos State) were observed communicating effectively about follow-up visits, and exit interview participants indicated knowing when to bring their child for their next vaccination. Caregivers in Ikorodu (Lagos State) were observed being told when to bring their child back for vaccination in only one out of six facility observations; however, during exit interviews 94% caregivers in Ikorodu reported knowing when to bring back the child for his/her next vaccination. We are unable to completely explain this discrepancy. As we did not determine if the caregiver was able to accurately indicate the date of the child’s next vaccination, we are unsure if caregivers did, in fact, know when they should return. It is possible that some caregivers may have indicated that they knew when to return but were mistaken. However, we believe it is more likely that caregivers simply understand the spacing between vaccinations, despite the fact that few of them were given this information during their session.

Though all caregivers in Kano State were told which vaccines their children were receiving, HCWs in Lagos State did not always share this information, which is a particular concern given that the right to this information is guaranteed under the Nigeria Patient Bill of Rights.^15^ A 2020 study of awareness and knowledge of the Patient Bill of Rights amongst physicians and patients concluded that awareness in both groups was low.^15^ Though we did not set out to assess observance of patient rights we suspect that awareness may be similarly low in Lagos State, suggesting that training of HCW and clarity on precisely how patient rights can be realised during the caregiver/HCW encounter is needed. In addition, methods for ensuring that caregivers are aware of their rights, and the rights of their children, should be explored.

All HCWs in Gezawa and Ungogo LGAs in Kano noted that a major strategy to improve vaccination coverage involves constant awareness and sensitisation through engaging community mobilisers, community leaders, religious leaders, and ward head leaders due to their influence within their communities. However, the exit interviews revealed that caregivers in both states typically obtain information about vaccination from HCWs which corroborates findings from other studies in Nigeria.^4,16,17^ Moreover, fear of AEFI is known to strongly predict UI leading Omoleke *et al* to conclude that effective engagement from HCW to caregiver has the potential to alleviate fear of AEFI and, in turn, reduce drop-out.^18^ Thus, skilled engagement - communicating the importance of vaccination, as well as the detection and management of AEFI - from HCW to caregiver (including via encounters within non-immunisation services) is likely a critical factor in increasing uptake of childhood vaccination amongst ZD children, and reducing UI due to drop-out.

Male caregivers expressed divergent views on childhood vaccination. Many of the male caregivers involved in the FGDs agreed that immunisation is effective and safe and, therefore, they supported vaccination of their children. However, some, particularly male caregivers in Kano, did not support vaccination because of previous experiences of side effects.

#### Poor Attitude of Health Care Workers

Caregivers reported encountering poor attitudes, poor interpersonal communication, and a lack of empathy from HCWs which mirror findings from other recent studies in Nigeria.^4,17,19^ Staff reported feeling demotivated when their allowance payments were not issued regularly. We believe it likely that this, and other factors relating to understaffing and the need to pay for transportation of vaccines, demotivates HCWs which may, in turn, impact the quality of the interaction between HCWs and caregivers. However, our hypothesis may lack nuance as a 2023 study of the drivers of childhood immunisation inequality suggested that disrespectful care (and eroded HCW motivation) resulted from distorted power relationships - that at once privilege and disempower HCWs – which, in turn, created, “a vicious cycle of negative power relations between health workers and clients”^19p. 9^. The study also observed that caregivers of low socioeconomic status (SES) were more likely to experience disrespectful care; a particularly concerning finding given that lower SES is associated with lower childhood vaccination coverage.^19,20^ One of the most common recommendations provided by caregivers, and indeed by respondents from other studies of HCW/client interactions in Nigeria,^16,21^ for improving services was addressing disrespectful and judgemental encounters with HCWs. However, it is important to acknowledge that though several studies have found that HWC attitudes impact childhood vaccination uptake, the majority of HCW interactions with caregivers are positive.^17,21^ It is also worth noting that 300 out of 302 (99%) caregivers in Kano, and 286 out of 308 (93%) in Lagos, indicated that they were satisfied with vaccination services suggesting that negative experiences with HCWs, whilst important to address, are either uncommon, or have little bearing on satisfaction with services. Finally, the difference between the high caregiver satisfaction reported in the exit interviews, and the many concerns raised during the FGDs is somewhat puzzling. However, we suggest that the different data collection approaches elicit information about the same underlying concept (i.e. caregiver satisfaction with services) but that this concept is abstracted at different levels (i.e. caregivers report overall satisfaction in the exit interviews, but are given the opportunity to reflect on and discuss specific challenges during the FGDs).

#### Low HCW Capacity to Carry out Planning Activities

Data from the KIIs indicate that staff in Kano State did not know how to estimate the population of children under one year. The KIIs also revealed that data are not used to plan outreach activities. These findings are similar to those of Ali *et al* who found that, “[t]he challenges and barriers to achieving high immunisation coverage in Nigeria include inadequate planning, poor implementation of planned activities, [and] lack of monitoring for action…”^22p. 147^.

Our findings also indicate that health facilities had sufficient vaccines to conduct immunisation sessions, and there were strategies in place to manage stockouts, such as obtaining vaccine stocks from neighbouring health facilities. This approach helped reduce missed opportunities, drop-outs, and dissatisfaction. However, some caregivers reported being turned away in the past due to stockouts. This implies that missed opportunities for vaccination due to stockouts may be responsible for some of ZD and UI children in the study population. We hypothesise that occasional stockouts may be due to gaps in administrative planning (e.g. poor forecasting), rather than vaccine unavailability in the country. A 2022 systematic review of medicine and supply chain challenges in Nigeria support our findings; the review identified several important challenges to supply of vaccines including: poor procurement; incomplete forecasting; poor data collection, use, and management; and poor reliability and availability of data for forecasting and decision making.^23^

#### Health and Safety Practices

In addition to health and safety issues relating to low staff numbers, observations from the facility observation, and feedback from the exit interviews suggest that whilst health and safety practices were generally good some improvements should be considered, particularly relating to waste disposal and handling of needles. Supportive supervision has been shown to improve service quality suggesting a straightforward solution to improving the health and safety of immunisation services in our study settings; however, it is important to note that supportive supervision has not been shown to be as effective in addressing structural issues (e.g. staffing numbers) which may, in part, be contributing to some of the observed quality issues.^24^

### Facility-Level Factors

#### Poor Facility Environment

Qualitative findings from the FGDs suggest that healthcare workers often exhibit a poor attitude including poor interpersonal communication, tardiness, and a lack of empathy. The facility environment was also sometimes described as unwelcoming, with caregivers highlighting restrictive and inconvenient opening times, and long waiting times. A 2019 study exploring reasons for missed opportunities for vaccination (MOV) in Kano determined that many of the MOVs can be attributed to factors outside the control of the caregiver.^25^ The MOV factors identified in this study closely mirror our findings and include: a lack of kindness and flexibility (particularly when a caregiver is late for immunisation services) from HCWs, and poor built environment of facilities (which may cause or worsen the discomfort of waiting outside for services)^14(p. 1.)^. Similarly, a study in Ogun State found that community members had a mostly unfavourable perception of health care facilities owing to the facility environment, infrastructure, and lack of equipment and supplies; “…this resulted in a reluctance of the community members to access care in their health facilities further reducing RI utilisation”.^26^ Recommendations from facility observations and from caregivers suggest that, taken together, multiple features of the HCW/caregiver interaction and the built environment contribute to creating an unwelcoming experience. Recommendations include: 1) ensuring staff are friendly, mature, organised, professional, polite, kind, and respectful; 2) improving facility ventilation, providing electricity and fans, providing chairs, increasing space, improving facility cleanliness; and 3) starting immunisation sessions earlier, reducing waiting times, and improving management of the queue (see Additional File 3 in the Supplemental Material).

### Programme Design Factors

#### Poor Service Integration or Low Uptake of Multiple Services

An important missed opportunity to incentivise vaccination is poor service integration and low uptake of other available services. Our findings from the exit interviews substantiate findings from the facility observation that suggest that vaccination services are often the only service accessed during the visit. A significant proportion of caregivers reported having had difficulty accessing the health facility, and several caregivers reported having been turned away when attending vaccination services in the past. Taken together this suggest that bundling services (or more strategic bundling of services) might be incentivising, particularly for those caregivers who might be disinclined to attend a follow-up visit given the difficulty accessing services, or the possibility of being turned away.

More information is needed about the best combination of services to offer on vaccination service days. Given that decisions about childhood vaccination are commonly made by male caregivers, pairing ANC services with immunisation services to encourage expectant mothers to vaccinate their children, may have limited impact on reducing ZD children. However, pairing immunisation services with another complementary service (i.e. another service targeted at caregivers with young children) may be more impactful in reducing drop-out by encouraging caregivers to make the sometimes-onerous journey to the health facility on vaccination days, even when there is a risk (or a perceived risk) of discovering that vaccination services are unavailable upon reaching the facility. This approach is consistent with the federal government’s ‘Primary Health Care Under One Roof’ policy which aims to improve service integration within primary health care services.^27^

#### Insufficient Staff Numbers to Carry out Vaccination Activities

Facility observation and KIIs indicate an insufficient number of staff to carry out vaccination activities (particularly in Lagos State) yet exit interviews with caregivers suggest very high satisfaction with vaccination services. It was not within the study scope to determine the cause of low staffing numbers, however, there are many possible causes including: absence due to sickness and/or demotivation, partial implementation of Nigeria’s primary health care or immunisation policies, the increase in health care facilities (without a corresponding increase in budget), low incentives for HCW and consequent staff turnover, and the reliance of the immunisation program on project staff whose contracts have ended. The relatively high number of reported staff shortages in Lagos may also be due, in part, to the considerable out-migration of HCWs in Nigeria.^28^ As emigration pressure is generally higher in the southern Nigeria it is possible that this may explain some of the staff shortages in Lagos State, when compared to Kano State.

Other indicators that HCW may be overstretched were not observed during facility observation: i.e. health information management materials were present, staff appeared skilled in carrying out vaccinations, and information about follow-up vaccinations was communicated to all caregivers in all but one instance. However, recommendations made by data collectors (following facility observations) and caregivers include increasing staff numbers. Finally, participants in a 2019 study of immunisation uptake in Ogun State were unanimous in their agreement that increasing the number of HCWs would have the effect of increasing vaccine availability.^17^

#### Lack of Resources for Transportation, Outreach Services, Demand Generation Activities

The KIIs suggest that outreach activities are subject to delays due to funding and lack of transportation. Some HCW reported having used their own funds to transport vaccines. Whilst some facilities had facility-level microplans, not all facility microplans were specifically used for following-up children who had missed a follow-up visit, or for reaching migrant populations. In addition, health facilities had no plan or budget for social mobilisation and community engagement.

### Limitations and Strengths of the Study

A key study limitation relates to our use of purposive sampling. Whilst our sampling approach ensured representation for a diverse study population, our study sample may not represent the population characteristics of Kano or Lagos States. However, the diversity of our sample has provided rich contextual information and, importantly, the views of male caregivers and caregivers of ZD children.

This study used a cross-sectional design; thus, we cannot determine to what extent programmes or services are improving or worsening. Whilst this was not an aim of our study it should be noted that in 2023 the Government of Nigeria committed to intensifying efforts to restore immunisation progress following the COVID-19 pandemic, including by strengthening health care workforces and improving health service delivery.^6^ As such, participants in our FGDs/KIIs may have been describing recent experiences that may no longer reflect challenges with immunisation services in their respective LGAs.

Facility observation, and perhaps to a lesser extent exit interviews, may have been subject to observation bias which may explain why we did not observe antagonistic behaviour from HCWs despite this being raised as a key concern by caregivers during FGDs. Finally, we did not collect information on religion or ethnicity and therefore cannot account for how these factors may impact the experience of immunisation services.

Ultimately, the strengths of our study lie in our use of mixed methods, the diversity of our study population, and the skill of our experienced data collection teams.

## Conclusions

Our study used mixed methods to describe immunisation services in Kano and Lagos States. Though there have been important recent improvements in service design and delivery, several important gaps remain. We suspect that many of the challenges we identified are interrelated (e.g. insufficient staff numbers and an uncomfortable facility environment, may contribute to poor interaction between healthcare workers and caregivers) and that addressing some of these challenges may be cost-effective by creating cascading improvements to services and, by extension, vaccination coverage. Regardless, a clear strategy for addressing the above challenges - developed in close collaboration with caregivers (including male caregivers), communities, and HCWs – is needed to reduce the number of ZD and UI children in Kano and Lagos States.

We agree with other researchers who have drawn attention to the importance of understanding the important socio-cultural differences between states, and their role in immunisation service provision and uptake.^5,16,18^ Though many challenges with immunisation services were identified in both states, solutions should ideally be tailored to each setting. Furthermore, targeted strategies to reach specific hard-to-reach populations (including conflict-affected, migrant, or extremely remote populations) may help to improve reach.

Finally, whilst our method did not seek to establish a link between service quality and immunisation uptake our FGDs strongly suggested that poor service quality in our study areas has frustrated efforts to improve vaccination coverage and equity. We believe that even modest improvements to service quality, particularly those that improve community perception of service, will improve coverage, reduce drop-out, and provide benefits to health services more broadly. We endorse the WHO recommendation that: “[a]t the point of service, immunization programmes need to implement evidence-based interventions that demonstrate improvement; […] incorporating the perspective of communities into the planning and delivery of immunization services is key to achieving equitable access to immunization and reaching underserved communities”^8p. 20^. We encourage regular evaluations to inform continuous improvement of immunisation programmes; evaluations co-designed with communities would likely offer the greatest insight.

## Supplemental Material

Supplemental Material - Assessment of Childhood Immunisation Services in Kano and Lagos States, Nigeria: A Mixed Methods Study Supplemental Material for Assessment of Childhood Immunisation Services in Kano and Lagos States, Nigeria: A Mixed Methods Study by Adekunle Ganiyu Salaudeen, Oladimeji Akeem Bolarinwa, Catherine R. McGowan, Drichi Daniel, Mervat Alhaffar, Kaja Abbas, Olatunde Adesoro, Tahlil Ahmed, Andrew Clarke, Sostine Makunja, Nada Abdelmagid, Tanimola Makanjuola Akande in Health Services Insights.

## Data Availability

The dataset supporting the conclusions of this article is included in the Supplemental Material.*

## Appendix

### List of Abbreviations

1YL: First year of life
AEFI: Adverse event following immunisation
AFP: Acute flaccid paralysis
ANC: Ante-natal care
BCG: Bacillus Calmette–Guérin vaccine
BeSD: Behavioural and social drivers
CHEW: Community health extension worker
DTP1: Diphtheria tetanus toxoid and pertussis (first dose)
DTP3: Diphtheria tetanus toxoid and pertussis (third dose)
EPI: Expanded programme on immunisation
FGD: Focus group discussions
HCW: Health care worker
ITN: Insecticide-treated nets
IWI: International wealth index
LGA: Local Government Authority
KII: Key informant interviews
MICS/NICS: Multiple Indicator Cluster Survey/National Immunization Coverage Survey
MOV: Missed opportunities for vaccination
NERICC: National Emergency Routine Immunization Coordination Center
NGO: Non-governmental organisation
OIC: Officer-in-charge
RI: Routine immunisation
SES: Socio-economic status
TBA: Traditional birth attendants
U5MR: Under five mortality rate
UI: under-immunised
VCM: Village/volunteer community mobilisers
VPD: Vaccine-preventable diseases
WHO: World Health Organization
ZD: Zero-dose.
