# Supplementary material for: Assessment of Childhood Immunisation Services in Kano and Lagos States, Nigeria: A Mixed-methods Study
Source: Health Serv Insights. 2026 Sep 17;19:11786329261477836. doi: 10.1177/11786329261477836 (PMC13724904; doi:10.1177/11786329261477836)
Supplement: Supplementary file 1 [file sj-pdf-1-his-10.1177_11786329261477836.pdf]

ADDITIONAL FILE 1: Facility observation results by state, and LGA*

	Gezawa		Ungogo		KANO		Alimosho		Ikorodu		LAGOS	
	Freq	Prop	Freq	Prop	Freq	Prop	Freq	Prop	Freq	Prop	Freq	Prop
How many health workers were present during the vaccination session?												
1-5	7	1.0	4	0.8	11	0.9	11	0.8	5	0.8	16	0.8
6-10	0	0.0	1	0.2	1	0.1	3	0.2	1	0.2	4	0.2
Were any community volunteers present and assisting the session?												
Yes	5	0.7	3	0.6	8	0.7	11	0.8	0	0.0	11	0.6
No	2	0.3	2	0.4	4	0.3	3	0.2	6	1.0	9	0.5
Are there enough staff available to run a session?												
Yes	6	0.9	4	0.8	10	0.8	4	0.3	4	0.7	8	0.4
No	1	0.1	1	0.2	2	0.2	10	0.7	2	0.3	12	0.6
Do you feel staff had skills to run a high-quality session?												
Yes	7	1.0	5	1.0	12	1.0	13	1.0	6	1.0	19	1.0
No	0	0.0	0	0.0	0	0.0	0	0.0	0	0.0	0	0.0
Do health sector staff appear polite and organized?												
Yes	6	0.9	5	1.0	11	0.9	13	1.0	4	0.7	17	0.9
No	1	0.1	0	0.0	1	0.1	0	0.0	2	0.3	2	0.1
Are vaccines stored at appropriate temperatures during the session?												
Yes	6	0.9	5	1.0	11	0.9	13	1.0	6	1.0	19	1.0
No	1	0.1	0	0.0	1	0.1	0	0.0	0	0.0	0	0.0
Is a register (or adequate recording tool) available at the session?												
Yes	7	1.0	5	1.0	12	1.0	13	1.0	6	1.0	19	1.0
No	0	0.0	0	0.0	0	0.0	0	0.0	0	0.0	0	0.0
Are vaccination/health cards available at the session?												
Yes	7	1.0	5	1.0	12	1.0	13	1.0	6	1.0	19	1.0

No	0	0.0	0	0.0	0	0.0	0	0.0	0	0.0	0	0.0
Is a tally sheet available at the session?												
Yes	7	1.0	5	1.0	12	1.0	13	1.0	6	1.0	19	1.0
No	0	0.0	0	0.0	0	0.0	0	0.0	0	0.0	0	0.0
Is a safety box available at the session?												
Yes	7	1.0	5	1.0	12	1.0	13	1.0	6	1.0	19	1.0
No	0	0.0	0	0.0	0	0.0	0	0.0	0	0.0	0	0.0
Do you have suggestions for improving session quality?												
Yes	3	0.4	5	1.0	8	0.7	11	0.9	3	0.5	14	0.7
No	4	0.6	0	0.0	4	0.3	2	0.2	3	0.5	5	0.3
Is each vaccine administered using the correct route of vaccination?												
Yes	7	1.0	5	1.0	12	1.0	12	1.0	6	1.0	18	1.0
No	0	0.0	0	0.0	0	0.0	0	0.0	0	0.0	0	0.0
Is each vaccine prepared using the correct diluent for the vaccine?												
Yes	7	1.0	5	1.0	12	1.0	12	1.0	6	1.0	18	1.0
No	0	0.0	0	0.0	0	0.0	0	0.0	0	0.0	0	0.0
Does the vaccinator touch or recap the needle?												
Yes	3	0.4	0	0.0	3	0.3	1	0.1	0	0.0	1	0.1
No	4	0.6	5	1.0	9	0.8	11	0.9	6	1.0	17	0.9
Are used needles disposed of in a proper safety box after each vaccination?												
Yes	7	1.0	5	1.0	12	1.0	11	0.9	6	1.0	17	0.9
No	0	0.0	0	0.0	0	0.0	1	0.1	0	0.0	1	0.1
Is the caregiver told when to bring the child back for vaccination?												
Yes	7	1.0	5	1.0	12	1.0	12	1.0	1	0.2	13	0.7
No	0	0.0	0	0.0	0	0.0	0	0.0	5	0.8	5	0.3
Is the caregiver told about potential adverse events following vaccination?												

Yes	5	0.7	5	1.0	10	0.8	9	0.8	3	0.5	12	0.7
No	2	0.3	0	0.0	2	0.2	3	0.3	3	0.5	6	0.3
If the caregiver asks any questions are they answered?												
Yes	5	0.7	4	0.8	9	0.8	9	0.8	4	0.7	13	0.7
No	2	0.3	1	0.2	3	0.3	3	0.3	2	0.3	5	0.3
Is each vaccination recorded in the child health card?												
Yes	7	1.0	5	1.0	12	1.0	11	1.0	6	1.0	17	1.0
No	0	0.0	0	0.0	0	0.0	0	0.0	0	0.0	0	0.0
Is each vaccination recorded in the clinic's registry?												
Yes	7	1.0	5	1.0	12	1.0	11	1.0	6	1.0	17	1.0
No	0	0.0	0	0.0	0	0.0	0	0.0	0	0.0	0	0.0
Any additional health services received today?												
Yes	5	0.7	0	0.0	5	0.4	6	0.5	4	0.7	10	0.6
No	2	0.3	5	1.0	7	0.6	6	0.5	2	0.3	8	0.4
Is the caregiver told which vaccines the child is receiving?												
Yes	7	1.0	5	1.0	12	1.0	9	0.8	5	0.8	14	0.8
No	0	0.0	0	0.0	0	0.0	3	0.3	1	0.2	4	0.2
Did the child receive appropriate vaccines												
Yes	7	1.0	5	1.0	12	1.0	12	1.0	6	1.0	18	1.0
No	0	0.0	0	0.0	0	0.0	0	0.0	0	0.0	0	0.0
Is there safe disposal of all sharp objects used during vaccination?												
Yes	7	1.0	5	1.0	12	1.0	12	1.0	6	1.0	18	1.0
No	0	0.0	0	0.0	0	0.0	0	0.0	0	0.0	0	0.0
Are all the wastes disposed appropriately after vaccination?												
Yes	7	1.0	4	0.8	11	0.9	10	0.8	6	1.0	16	0.9
No	0	0.0	1	0.2	1	0.1	2	0.2	0	0.0	2	0.1

*Not all checklist items were recorded for some facilities thus the denominator does not always reflect the number of facilities surveyed.

ADDITIONAL FILE 2: Exit interview results by state, and LGA*

	Gezawa		Ungogo		KANO		Alimosho		Ikorodu		LAGOS	
	Freq	Prop	Freq	Prop	Freq	Prop	Freq	Prop	Freq	Prop	Freq	Prop
What services did you receive today?												
Vaccination	154	0.99	147	1.00	301	1.00	155	0.99	153	1.00	308	1.00
Growth monitoring	1	0.01	0	0.00	1	0.00	117	0.75	150	0.98	267	0.86
Curative care	1	0.01	0	0.00	1	0.00	5	0.03	6	0.04	11	0.04
Other	2	0.01	0	0.00	2	0.01	3	0.02	1	0.01	4	0.01
Do you have child's health vaccination card?												
Yes	155	1.00	147	1.00	302	1.00	156	1.00	153	1.00	309	1.00
No	0	0.00	0	0.00	0	0.00	0	0.00	0	0.00	0	0.00
Is the child up to date on vaccinations?												
Yes	139	0.90	137	0.93	276	0.91	151	0.97	145	0.95	296	0.96
No	16	0.10	10	0.07	26	0.09	5	0.03	8	0.05	13	0.04
Is it difficult to reach the health facility?												
Yes	50	0.32	66	0.45	116	0.39	32	0.21	39	0.25	71	0.23
No	104	0.68	81	0.55	185	0.61	124	0.79	114	0.75	238	0.77
Do you have any concerns about the vaccine?												
Yes	17	0.11	11	0.07	28	0.09	26	0.17	20	0.13	46	0.15
No	138	0.89	135	0.92	273	0.90	124	0.79	133	0.87	257	0.83
Don't know	0	0.00	1	0.01	1	0.00	6	0.04	0	0.00	6	0.02
Do you believe vaccines protect your child from disease?												
Yes	155	1.00	146	0.99	301	1.00	153	0.98	153	1.00	306	0.99
No	0	0.00	1	0.01	1	0.00	2	0.01	0	0.00	2	0.01

Don't know	0	0.00	0	0.00	0	0.00	1	0.01	0	0.00	1	0.00
Have you ever refused vaccinations for your child?												
Yes	18	0.12	16	0.11	34	0.11	15	0.10	5	0.03	20	0.06
No	137	0.88	131	0.89	268	0.89	141	0.90	148	0.97	289	0.94
Don't know	0	0.00	0	0.00	0	0.00	0	0.00	0	0.00	0	0.00
Do you know when to bring child for next vaccination?												
Yes	152	0.99	143	0.97	295	0.98	152	0.97	144	0.94	296	0.96
No	2	0.01	4	0.03	6	0.02	4	0.03	9	0.06	13	0.04
Were you ever turned away for any vaccination and told to come back later?												
Yes	21	0.13	17	0.12	38	0.12	21	0.14	10	0.07	31	0.10
No	135	0.87	130	0.88	265	0.88	134	0.86	143	0.93	277	0.90
If Yes specify												
Too few kids	1	0.01	0	0.00	1	0.00	3	0.02	2	0.01	5	0.02
Staff not available	1	0.01	0	0.00	1	0.00	0	0.00	0	0.00	0	0.00
Child not correct age	8	0.05	2	0.01	10	0.03	0	0.00	0	0.00	0	0.00
Child was too ill	1	0.01	0	0.00	1	0.00	0	0.00	0	0.00	0	0.00
No vaccine	6	0.04	14	0.10	20	0.07	9	0.06	5	0.03	14	0.05
Other	4	0.03	1	0.01	5	0.02	9	0.06	3	0.02	12	0.04
How do you hear about immunization services?												
Radio/TV/ poster	59	0.38	69	0.45	128	0.42	33	0.21	55	0.36	88	0.28
Community groups	68	0.44	45	0.31	113	0.37	27	0.17	12	0.08	39	0.13
Village	23	0.15	62	0.42	85	0.28	4	0.03	10	0.07	14	0.05

volunteers												
Health workers/nurse	92	0.59	85	0.58	177	0.59	108	0.69	120	0.78	228	0.74
Phone message	2	0.01	1	0.01	3	0.01	1	0.01	1	0.01	2	0.01
Family	52	0.34	52	0.35	104	0.34	86	0.55	53	0.35	139	0.45
Friends	31	0.20	32	0.22	63	0.21	37	0.24	18	0.12	55	0.18
Other	6	0.04	6	0.04	12	0.40	5	0.03	3	0.02	8	0.03
If you had another baby today, would you want your baby to get all recommended vaccinations?												
Yes	155	1.00	146	0.99	301	1.00	154	0.99	153	1.00	307	0.99
No	0	0.00	1	0.01	1	0.00	2	0.01	0	0.00	2	0.01
Don't know	0	0.00	0	0.00	0	0.00	0	0.00	0	0.00	0	0.00
In general, are you satisfied with vaccinations services?												
Yes	153	0.99	147	1.00	300	0.99	147	0.95	139	0.91	286	0.93
No	2	0.01	0	0.00	2	0.01	8	0.05	14	0.09	22	0.07

*Caregivers were not required to respond to questions; thus the denominator does not always reflect the number of caregivers surveyed.

ADDITIONAL FILE 3: Facility observation and caregiver suggestions for improving service design and delivery

	Facility observation	Caregivers
2. Human resources management	• Employ cleaners for the health facilities<br>• Provide refresher training for HCWs<br>• Employ more than one vaccinator per health facility	• Staff should be friendly, mature, organised, professional, polite, kind and respectful<br>• There should be more than two staff per facility
3. Vaccine supply, quality and logistics	• Increase vaccine storage facilities	• Supplies - vaccines, chairs, pampers, food, transport money, weighing scale
4. Service delivery	• Increase space at the facility and provide chairs<br>• Integrate other services; e.g. insecticide-treated nets (ITNs)<br>• Better management of queue	• Services- growth monitoring, deworming medicine & vitamin, follow queue and free & continuous services<br>• Health facilities - renovation, improve ventilation, provide electricity & fans, increase spaces, clean, immunisation equipment & maintenance, free services<br>• Time management - Early start of sessions and reduced waiting time
7. Demand generation	• Improve communication skills of vaccinators	• Awareness for fathers, awareness through churches, mosques & schools and awareness about immunisation provided by nearby facilities<br>• incentives,
